# Diversity and Seasonal Abundance of *Culicoides* (Diptera: Ceratopogonidae) in Tengchong County of Yunnan, China

**DOI:** 10.3390/insects16080780

**Published:** 2025-07-30

**Authors:** Yi-Nan Wang, Ying-Liang Duan, Zhan-Hong Li, Jia-Ming Deng, Xing-Nan Sun, Xue-Ying Shen, An-Xi Yang, Shi-Long Li

**Affiliations:** 1 Yunnan Tropical and Subtropical Animal Virus Diseases Laboratory, Yunnan Animal Science and Veterinary Institute, Kunming 650224, China; wangyinan0546@163.com (Y.-N.W.); dy081lzh@163.com (Z.-H.L.); 2 Key Laboratory of Transboundary Animal Diseases Prevention and Control (Co-Construction by Ministry and Province), Ministry of Agriculture and Rural Affairs, Kunming 650224, China; 3 Center for Animal Disease Control and Prevention, Tengchong 679100, China; 18987516163@163.com (J.-M.D.); 18187581304@163.com (X.-N.S.); yntcsxy@163.com (X.-Y.S.); 18788007006@163.com (A.-X.Y.); 4 Animal Husbandry and Veterinary Station of Mingguang Town, Tengchong 679103, China; mgsyzlsl@163.com

**Keywords:** *Culicoides*, diversity, seasonality, *C. tainanus*, *C. obsoletus*, *C. fenggangensis*, vector, *cox1*, Tengchong, China

## Abstract

*Culicoides* (Diptera, Ceratopogonidae) are small biting midges and are known as vectors for many arboviruses, including bluetongue virus (BTV) and epizootic hemorrhagic disease virus (EHDV). Tengchong County of Yunnan Province, China, which borders Myanmar, has many private farms with goats, sheep, and cattle. To estimate the risk of *Culicoides*-borne viral diseases such as bluetongue (BT) and epizootic hemorrhagic disease (EHD) in this area, an investigation of the diversity and abundance of *Culicoides* in Tengchong between May 2024 and April 2025 was performed. As a result, 70 collections totaling approximately 93,000 *Culicoides* were carried out at five farms (cattle + Asian buffaloes, goats, and sheep, respectively). Nineteen species were identified, and eight potential cryptic species were found. A total of 13 *cox1* sequences and 4 *28S* sequences for 13 specimens were generated. The most dominant species were Obsoletus (44.1%), *C. homotomus* (23.3%), and *C. arakawae* (12.9%) at the bovine farm; *C. tainanus* (68.0%), *C. orientalis* (12.6%), and *C. newsteadi* (Asia) (6.3%) at the goat farm; and *C. tainanus* (73.6%), *C. fenggangensis* (7.3%), and *C.* sp. nr *palpifer* (6.3%) at the sheep farm. In this investigation, *C. tainanus*, Obsoletus, and *C. orientalis* were the most dominant potential BTV vectors, and the period between July and October may be the main period for epidemics of *Culicoides*-borne viruses in Tengchong.

## Simple Summary

Biting midges, *Culicoides*, display a great variety and are widely distributed in tropical and temperate zones. In addition, some *Culicoides* species are vectors of arboviruses. This study reports on the diversity and seasonal abundance of *Culicoides* in Tengchong County of Yunnan, China, between May 2024 and April 2025. This study offers valuable reference data on the taxonomy and diversity of *Culicoides* and the epidemiology of *Culicoides*-borne viruses in this area.

## 1. Introduction

*Culicoides* (Diptera: Ceratopogonidae) are small biting midges and are known as vectors for many arboviruses, including African horse sickness virus (AHSV), Akabane virus (AKAV), bluetongue virus (BTV), and epizootic hemorrhagic disease virus (EHDV) [1]. At least 1373 *Culicoides* species, barring fossil species, have been recorded worldwide and placed into 34 subgenera and 38 groups [2], with nearly 400 species recorded in China [3,4,5]. The species directory continues to steadily expand approximately every 5 years [2,3,6] as new regions are explored, and *Culicoides* species composition is expressively different between continents [4,7,8]. Compared to other hematophagous midges, such as *Leptoconops* Skuse and *Lasiohelea* Kieffer [9], most *Culicoides* species possess significant and distinctive morphological features, making them especially suitable for diversity research.

The *Culicoides* life cycle includes four stages (i.e., egg, larva, pupa, and adult), and the duration between eggs and F1 adults is approximately one month under laboratory conditions [10]. The pupae hatch into adults under proper humidity and temperature [11], and the female adults need animal blood to nurture their eggs after mating [12]. Because current data suggest that *Culicoides* are unable to transovarially transmit *Orbivirus* such as BTV to their offsprings [13,14], newly emerged midges are supposed to be free of arbovirus. Therefore, permissive adult *Culicoides* can be only infected through viremia blood meal and become arbovirus carriers. Thus, blood-fed, gravid, and parous females, rather than nulliparous females, may carry arbovirus.

Ruminants such as cattle, goats, and sheep are common blood hosts for many *Culicoides* species [15,16,17], but some, such as *Culicoides arakawae* (Arakawa) and *C. guttifer*, have a preference for avifauna [16,17,18] and *C. anophelis* extends the host range to mosquitoes [19]. As most adult *Culicoides* are mainly active at dusk and sunrise, light traps are usually operated overnight, including sunset and sunrise [7,20].

Written morphological descriptions accompanied by hand-drawn sketches were traditionally used for defining novel *Culicoides* species. The former may disregard important features for a species, and the latter usually creates morphological deviations. After that, the differences in usage of morphologic keys between Chinese and occidental taxonomy systems [4,7] also created barriers to a unified taxonomy of *Culicoides*. Morphological species descriptions are usually back up by museum voucher specimens which is not always possible with genetic identification. Therefore, genetic data has become a very useful auxiliary method to identify or clarify *Culicoides* species. The cytochrome c oxidase subunit 1 (*cox1*) and ribosomal RNA (rRNA) locus, including *18S rRNA*, *28S rRNA*, internal transcribed spacer (*ITS*), and external transcribed spacer (*ETS*), are common DNA regions used to identify animal species [21,22,23]. The *cox1* sequence is the primary gene barcode for insect classification [24,25,26,27].

Tengchong County is located in the west of Yunnan Province, China, and borders Myanmar, in where four arboviral diseases (dengue, Chikungunya, Japanese encephalitis virus infection, and Zika virus disease) receive much attention [28]. Especially Mingguang Town of Tengchong County, which borders Myanmar directly, is known for sheep and goat breeding. A total of 227.3 thousand bovine and 75.1 thousand goats + sheep were recorded by the end of 2024 in Tengchong, including 29.1 thousand bovine and 18.1 thousand goats + sheep in Mingguang Town. Although there is no report of animal arbovirus disease in Tengchong, arboviruses circulations are widespread in Yunnan Province. As the investigation of the diversity and abundance of *Culicoides* is helpful in estimating the risks of disease outbreaks such as bluetongue disease (BT), we attempted to investigate the diversity and abundance of *Culicoides* at bovine-, goat-, and sheep-breeding farms, respectively, in Tengchong County between May 2024 and April 2025. We also published the *cox1* and *28S* sequences for a few interesting specimens.

## 2. Materials and Methods

### 2.1. Collection of Biting Midges

**Figure 1:**
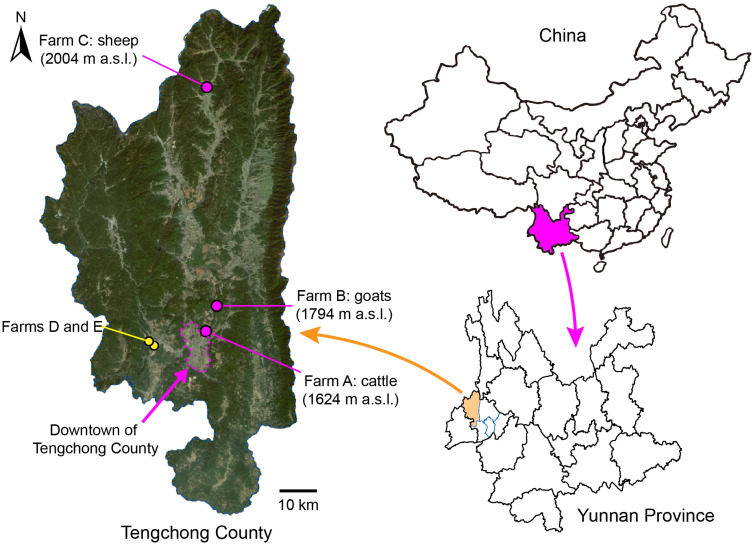
Light trap collecting sites for *Culicoides* midges in Tengchong County of Yunnan Province, China, between May 2024 and April 2025. The exact locations of the three monitoring sites (farms A, B, and C) are marked by pink dots, and their altitudes (meters above sea level, m a.s.l.) are labeled. Two minor collecting sites (farms D and E) are marked by yellow dots. The relief map is from Baidu Map.

Three farms, breeding bovines (cattle and Asian buffaloes), goats, and sheep, respectively, in Tengchong County of Yunnan Province, China, were identified as collection sites for midges (Figure 1, Table 1). Biting midges were collected once every two weeks between May 2024 and April 2025, with two additional collections conducted at two other farms during initial monitoring site selection (Table 1). Bovines on farm D were in the pens all the time, while all the animals on farms A, B, C, and E grazed in natural meadows in the morning and returned to pens before sunset every day. The numbers of animals on these farms remained relatively stable during the study period. A battery-powered UV-light trap (Yaoyu electronics Co., Ltd., Zhangzhou, China) coupled with a dry gauze bag was hung in the livestock pens (about 1.8 m above the ground) on each farm to collect midges from approximately 5:00 pm (before sunset) to 9:00 am the following day. Retrieved gauze bags were kept at −20 °C for approximately 15 min to immobilize the midges, and the collections were screened once using a steel strainer (mesh size = 4 mm) to remove large insects. Subsequently, the midges were kept in 100% ethanol at 4 °C until sorting.

### 2.2. Sorting the Culicoides

*Culicoides* species were identified and sorted according to the morphological keys of Wirth and Hubert [7] and Yu et al. [4], and were sorted into four categories (i.e., male, blood-fed female, parous + gravid female with blood digested, and nulliparous female) (Figure S1). Dyce’s method was used to distinguish between parous and nulliparous females [29]. Sorted midges were counted, and the rates of categories were calculated as follows: (1) male rate = male/total; (2) blood-fed rate = blood-fed females/total females; (3) parous + gravid rate = (parous + gravid) females/(parous + gravid + nulliparous) females; (4) nulliparous rate = 1 − (parous + gravid rate). Usually, all the specimens from each collection were sorted and counted, but if a collection was too big (approximately > 1000 *Culicoides*), only an aliquot of midges was dealt with. The proportion used for counting was calculated using the volumes of sedimentary insects in 100% ethanol. Sorted midges were kept in 75% ethanol at 4 °C until use.

### 2.3. Extracting DNA from Midges

A few specimens were individually allocated to PCR tubes and digested by 40 μL of tissue lysis buffer (TIANGEN, Tiangen Co., Beijing, China), at 30 °C overnight [30]. An aliquot of 30 μL lysate for each sample was submitted to purify DNA using a MagMAX^TM^-96 Viral RNA Isolation kit (Ambion, Thermo Fisher Scientific, Waltham, MA, USA) and MagMAX^TM^ Express-24 machine (Ambion) according to the manufacturer’s instructions. DNA was diluted using 50 μL of elution buffer and kept at −20 °C until use.

### 2.4. Mounting Specimens

Digested specimens were washed with water and mounted on slides [31]. Briefly, washed specimens were dehydrated using 75% ethanol for 10 min, 85% ethanol for 10 min, and 100% ethanol for 3–5 h. They were incubated in a 1:1 (*v*/*v*) ethanol-clove oil mixture for 1 day, followed by 100% clove oil for at least 24 h. Prepared specimens were cut into four parts (head, thorax, wing, and abdomen) and mounted on slides using a neutral balsam (#E675007, BBI Co., Ltd., Shanghai, China) and small cover glasses. Photos were taken using an Olympus^®^-CX31 microscope (Olympus Co., Tokyo, Japan) coupled with a BioHD^®^-FluoCa camera (Shanghai Fugai Optical Technology Co., Ltd., Shanghai, China).

### 2.5. Sequencing

Fragments of *cox1* and *28S rDNA* were amplified by high-fidelity enzyme using specific primers, respectively [30,32,33]. Briefly, 5 μL of DNA was added to 25 μL of PCR reaction solution made using PrimeSTAR^®^HS reagent (#R040A, Takara, Dalian, China) and primers according to the manufacturer’s instructions, and the *cox1* and *28S rDNA* were amplified by PCR as described by Duan et al. [30]. The PCR products were sent to Kunming Shuoqing Biological Technology Company (Kunming, China) for sequencing. A total of 13 *cox1* sequences and 4 *28S* sequences were acquired and uploaded to GenBank of National Center for Biotechnology Information (NCBI). The best-matched sequences on GenBank for these sequences were searched by BLAST of NCBI, respectively (https://blast.ncbi.nlm.nih.gov/Blast.cgi?PROGRAM=blastn&PAGE_TYPE=BlastSearch&LINK_LOC=blasthome, accessed on 27 May 2025).

### 2.6. Weather Data

The historical data of daily high temperature, low temperature, and weather for Tengchong County were acquired online [34]. To estimate daily precipitation quantitatively, rainfall scores were designed as 12 points for rainstorms or snowstorms, 9 points for heavy rain or snow, 4 points for moderate rain or snow, 2 points for light rain or snow, 0.4 points for overcast skies or fog, and 0 points for sun or clouds. If two weather regimes were recorded a day, the score was the average of the two cases. Photo period and humidity were not considered, since the duration of sunshine was nearly identical across all collection sites, and within-site humidity varied significantly within a day.

### 2.7. Analysis for the Dynamics of Midge Abundance

The high and mid temperatures on the day the traps were set, and the low temperature on the collection day were used to reflect the heat for a collection. The high and mid temperatures on the day the traps were set affected midge abundance, and approximately 5:00 a.m. on the collection day was the coldest time during the trapping. The average rain scores for 2, 5, and 10 days just before the collection date were also calculated. The prepared numeric and alphabetic string data were listed in a spreadsheet for big data analysis. Data of interest were used to construct graphics in the R program 4.5.0 [35] using the ggplot2 3.5.2 and gcookbook 2.0.1 packages according to the developers’ instructions [36].

## 3. Results

### 3.1. Diversity of Culicoides

A total of 70 collections containing approximately 93,000 biting midges were carried out at three monitoring sites and two additional farms (Tables S1 and S2). While the lowest numbers of midges were collected in the 24 collections made at the site (farm A) closest to downtown, the highest numbers were collected in the 20 collections made at the most distant site (farm C) from the downtown area (Figure 1, Table S1).

**Figure 2:**
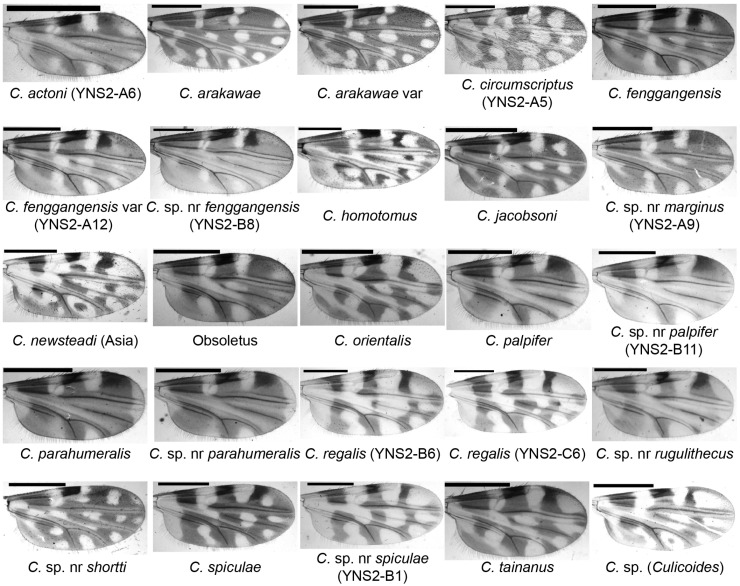
Wing patterns of *Culicoides* species collected by UV-traps in Tengchong County of Yunnan Province, China, between May 2024 and April 2025. The wing photos of female specimens are shown and their species names are labeled. The sample IDs for a few specimens by *cox1* sequence are labeled in brackets. Scale bar = 0.5 mm.

**Figure 3:**
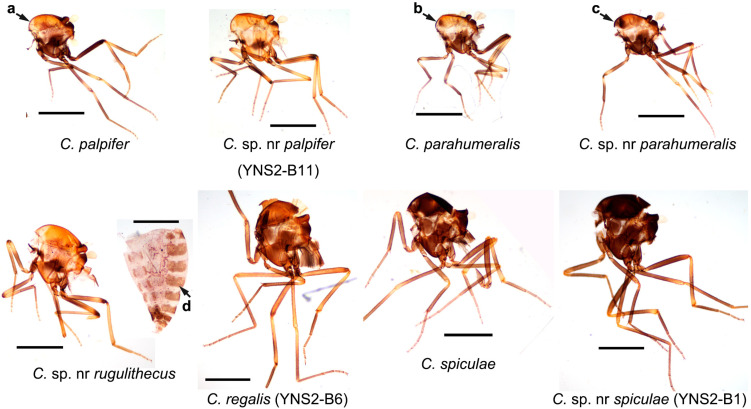
Thorax patterns of nine *Culicoides* species collected by UV-traps in Tengchong County of Yunnan Province, China, between May 2024 and April 2025. The thorax photos of eight female specimens and one abdomen photo are shown. The species names are labeled, and the sample IDs for a few specimens by *cox1* sequence are labeled in brackets. The major features for discussion are labeled with four arrows: a, no dot; b, three small brown dots; c, three large and contacted brown dots; d, relatively dark abdomen. Scale bar = 0.5 mm.

At least 19 known species, 8 potential cryptic species, and several unidentified species belonging to nine subgenera and one group were found (Table 2 and Table S2). The wing patterns of representative female specimens (Figure 2) and the thorax patterns of eight specimens (Figure 3) are shown. The five dominant species at farm A (bovine) were Obsoletus (Meigen) (44.1%), *Culicoides homotomus* Kieffer (23.3%), *C. arakawae* (12.9%), *Culicoides tainanus* Kieffer (7.3%), and *Culicoides fenggangensis* Liu & Hou (3.8%) (Table 2). The five dominant species at farm B (goats) were *C. tainanus* (68.0%), *Culicoides orientalis* Macfie (12.6%), *Culicoides newsteadi* Austen (Asia) (6.3%), *Culicoides* sp. nr *parahumeralis* Wirth & Hubert (2.9%), and *C. fenggangensis* (2.7%) (Table 2), and the five dominant species at farm C (sheep) were *C. tainanus* (73.6%), *C. fenggangensis* (7.3%), *Culicoides* sp. nr *palpifer* Das Gupta & Ghosh (6.3%), *Culicoides palpifer* Das Gupta & Ghosh (2.9%), and *C. orientalis* (2.2%) (Table 2). Contrarily, there were four species with a single midge only, i.e., *Culicoides actoni* Smith (farm E), *Culicoides hui* Wirth & Hubert (farm C), *Culicoides oxystoma* Kieffer (farm A), and *Culicoides parabubalus* Wirth & Hubert (farm B) (Table 2 and Table S2). In addition, only two specimens of *Culicoides circumscriptus* Kieffer were found at farm E (Table S2). After that, *C. homotomus* was only found at farm A. Also, there were three species (i.e., C. sp. nr *palpifer*, *Culicoides regalis* Majumdar & Das Gupta, and *Culicoides* sp. nr *shortti* Smith & Swaminath) and a mixed group (*Hoffmania* spp. with pale thorax) only appeared at the sheep farm (farm C) (Table 2).

A total of 13 *cox1* sequences (Table 3) and 4 *28S* sequences (NCBI: PV643096–PV643099) (Table S3) for seven or eight species (i.e., C. *actoni*, *C. circumscriptus*, *C. fenggangensis*, *Culicoides.* sp. nr *fenggangensis*, *Culicoides* sp. nr *marginus* Chu, *C.* sp. nr *palpifer*, *C. regalis*, and *Culicoides* sp. nr *spiculae* Howarth) were acquired and deposited in GenBank of NCBI.

### 3.2. Seasonal Abundance

**Figure 4:**
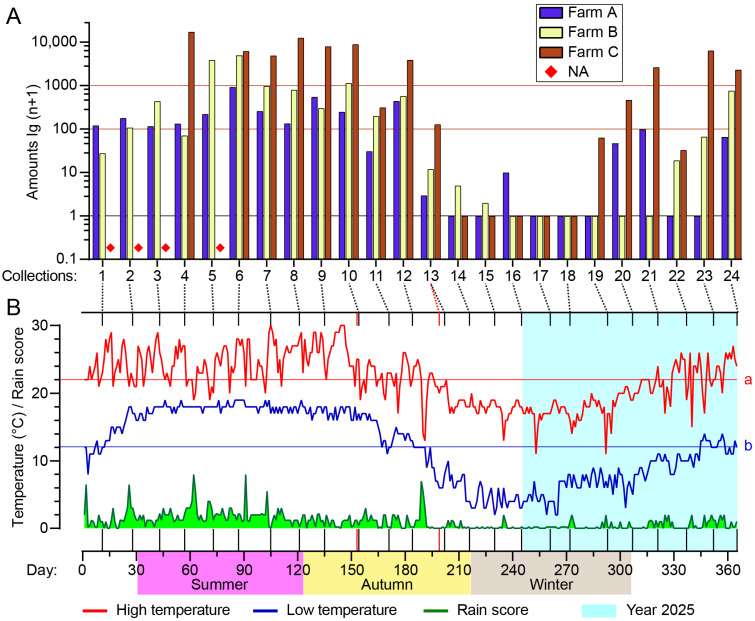
Seasonal abundance of *Culicoides* in Tengchong County of Yunnan Province, China, between May 2024 and April 2025. *Culicoides* were collected by UV-traps. (**A**) Total numbers of *Culicoides* for all collections (four missing collections are marked with a ◆) from the three monitoring sites. (**B**) Daily high temperature (red line), low temperature (blue line), and rain score (green area) of Tengchong County for 365 days between 1 May 2024 (1) and 30 April 2025 (365) are shown, and the average annual high (line a, 22.0 °C) and low (line b, 12.1 °C) temperatures are labeled. The 24 batches of collections from bovine and goat farms are marked by vertical lines; two red vertical lines mark the nonsynchronous collections at the goat farm.

Tengchong County is warm and rainy in summer and cool and dry in winter; its mean annual high and low temperatures are 22.0 °C and 12.1 °C, respectively (Figure 4B). On about half the days of the year, the daily low temperature is above the annual average. However, the actual temperatures at farm C (sheep) may be slightly lower than the official data used due to its higher altitude (2004 m a.s.l.) compared to the average altitude of Tengchong County.

*Culicoides* appeared between spring and mid-autumn (between March and the first half of November) by and large and reached a peak season of approximately 120 days between July and October (4th–12th collections), while hardly any midges were collected in late autumn and winter (Figure 4, Table S1).

**Figure 5:**
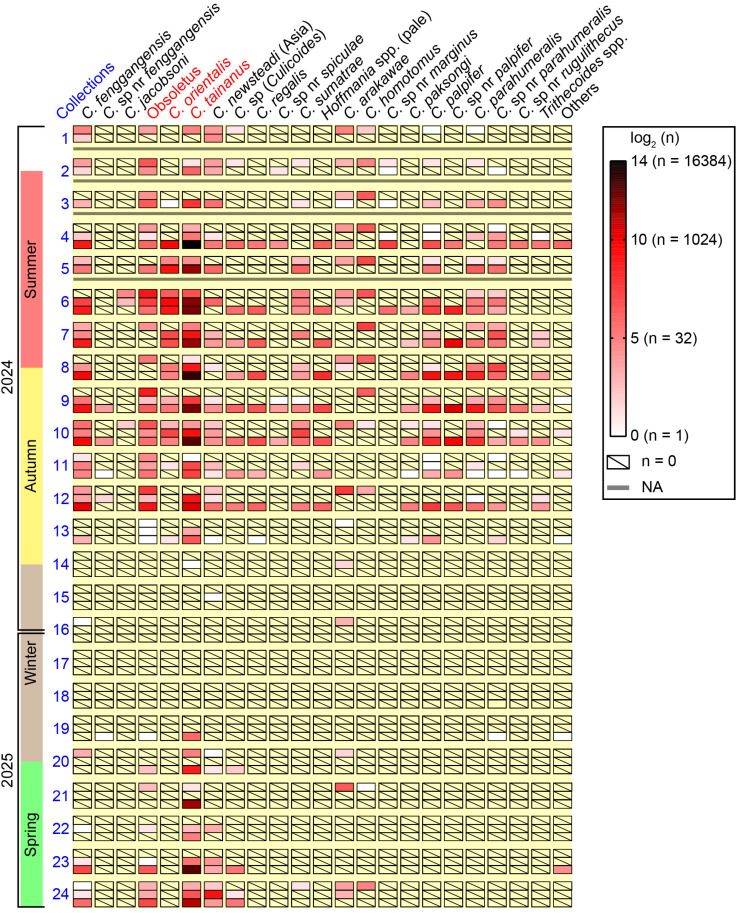
Amounts of major *Culicoides* species collected by UV-traps at the three monitoring sites in Tengchong County of Yunnan Province, China. The amounts of 23 groups of *Culicoides* in every collection from the three monitoring sites between May 2024 and April 2025, were shown by heat map according to the logarithm (log2) of amounts. Major potential BTV vectors were marked by red color. *Hoffmania* spp. (pale) represented a group of *Hoffmania* species with faint brown thorax, and “others” were a few unclassified *Culicoides* specimens. For each batch of collections, the up row, middle row and lower row represented the collections from farm A (bovine), farm B (goats), and farm C (sheep), respectively.

The seasonal abundances of most species at the three monitoring sites between May 2024 and April 2025 are shown in a heat map (Figure 5). *Culicoides tainanus* was obviously the most dominant species, while *C. homotomus* was one of the dominant species at farm A (bovine) and did not appear on the other two farms. The *Hoffmania* species with faint brown thorax (i.e., C. *regalis* and the group of unidentified *Hoffmania* spp.) and 4 *Trithecoides* species (i.e., *C. palpifer*, *C.* sp. nr *palpifer*, *Culicoides parahumeralis* Wirth & Hubert and *C.* sp. nr *parahumeralis*) were exuberant at the sheep farm between midsummer and mid-autumn. It was suggested that *C. fenggangensis*, Obsoletus, *C. tainanus*, *C. newsteadi* (Asia), *Culicoides* sp. (*Culicoides*) and *C. arakawae* are relatively cold-resistant species (Figure 5). 

### 3.3. Effects of Heat and Rainfall on Midge Abundance

**Figure 6:**
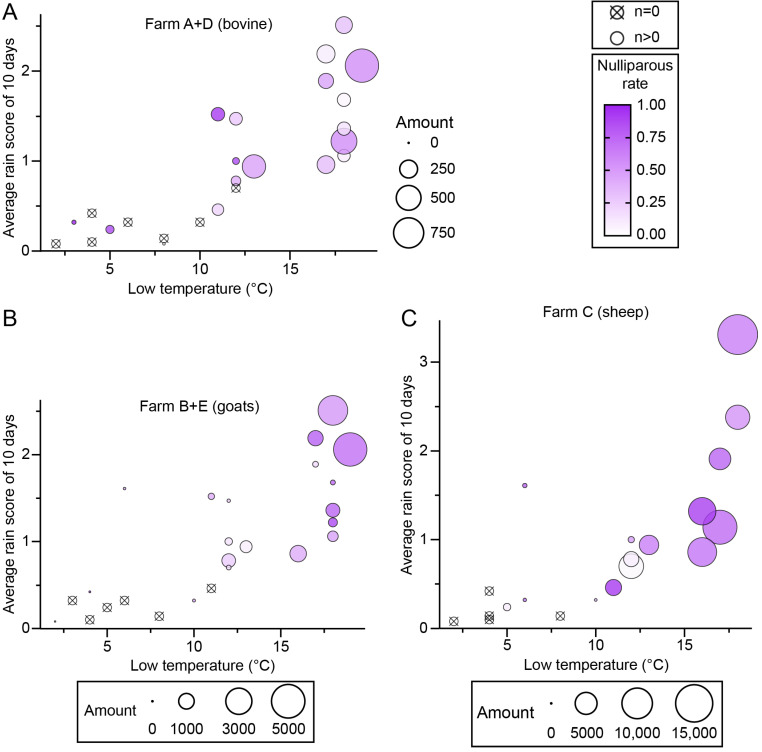
The effects of temperature and rainfall on nulliparous *Culicoides*. *Culicoides* were collected by UV-traps in Tengchong County of Yunnan Province, China, between May 2024 and April 2025. The relativities among the minimum temperature on the collection day (x-axis), average rain score for 10 days before the collection day (y-axis), the size of collection (area of circle), and the nulliparous rate (color gradation) are shown by scatter diagrams. Collections from (**A**) bovine farms (farms A and D), (**B**) goat farms (farms B and E), and (**C**) the sheep farm (farm C).

An analysis of the effects of heat and rainfall on midge population (amount and nulliparous rate) was performed using the average rain scores of 2 days, 5 days, and 10 days before the collection day and the high, mid, and low temperatures during the trapping. Among the results, midge amounts showed the best regularity with low temperature and an average rain score of 10 days (rain points/day) (Figure 6, Supplementary S1). A minimum temperature of 10 degrees (°C) and an average score of 0.5 points were apparently the threshold for adult midge activity, and a temperature above 15 °C and 1 point were ideal conditions for adult emergence (Figure 6). The nulliparous rate was roughly proportional to the low temperature and average rain score of 10 days, except for farm A (Figure 6).

### 3.4. Midge Status

**Figure 7:**
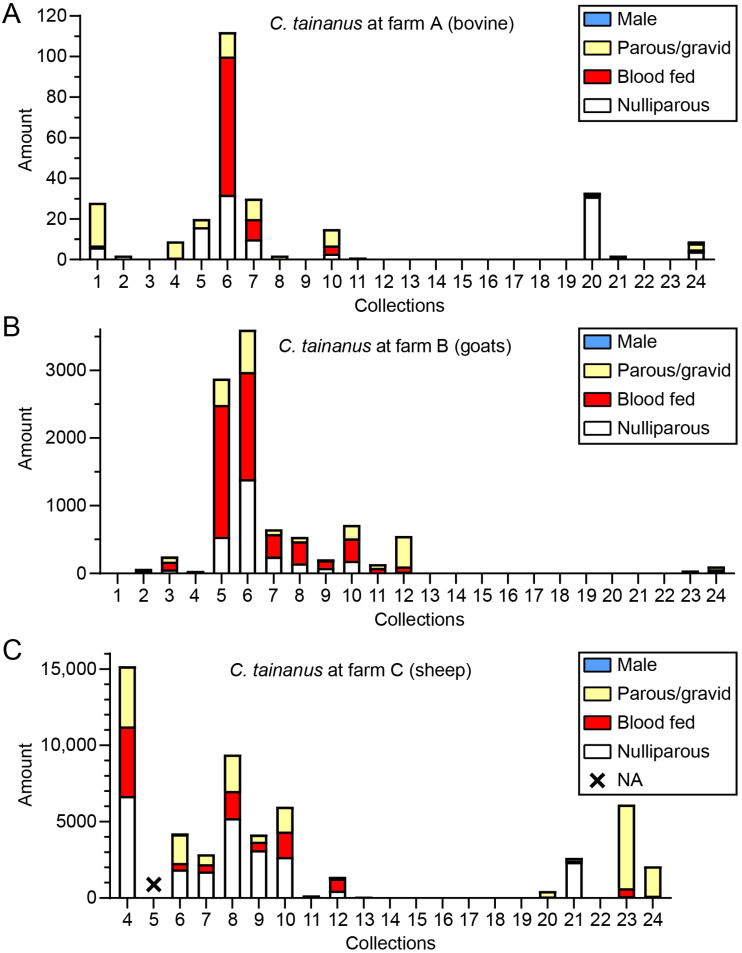
The status composition of *C. tainanus* collected by UV-traps in Tengchong County of Yunnan Province, China, between May 2024 and April 2025. The numbers of the four midge categories (males, parous/gravid females without blood meal, blood-fed females, and nulliparous females without blood meal) of *C. tainanus* in every collection from the three monitoring sites (**A**–**C**) are shown. NA = Not available collection.

The adult specimens were classified into four statuses (i.e., male, nulliparous female, blood-fed female, and parous/gravid female). The status composition of *C. tainanus*, Obsoletus, and *C. orientalis*, which were the dominant potential BTV vectors in this investigation, was determined. For *C. tainanus*, very few males were found in three collections; the blood-fed rates were high (>50%) in most collections at farms A and B in summer (Figure 7). For Obsoletus, no males were found, while the amounts and the blood-fed rates were very volatile in various collections during summer. The blood-fed rates were high at farm C (Figure S2). For *C. orientalis*, no males appeared either, and the blood-fed rates were less than 50% (Figure S3).

## 4. Discussion

This study represents the first report on the diversity and seasonal abundance of *Culicoides* in Tengchong County of Yunnan Province, China. At least 26 species (*C. parahumeralis* and *C.* sp. nr *parahumeralis* might be the same species) were found. Some specimens of uncertain species or possible new species/subspecies collected in this study were sequenced. A total of 13 specimens belonging to two known species (i.e., C. *actoni* and *C. circumscriptus*), two suspected species (i.e., *C. fenggangensis* and *C. regalis*), and 4 potential cryptic species (i.e., *C.* sp. nr *fenggangensis*, *C.* sp. nr *marginus*, *C.* sp. nr *palpifer*, and *C.* sp. nr *spiculae*) were sequenced and uploaded to GenBank (Table 3 and Table S3).

Many proven or potential BTV vectors, such as *C. actoni*, *C. imicola* Kieffer, *Culicoides jacobsoni* Macfie, *C. obsoletus*, *C. orientalis,* and *C. tainanus,* belong to the subgenus *Avaritia* [1,37,38,39]. In this investigation, *C. tainanus,* followed by Obsoletus and *C. orientalis,* were the most common potential BTV vectors in Tengchong County. *Culicoides tainanus* is widespread in East Asia [31,40,41] and proved to be a BTV carrier in Japan [40] and China [38], and *C. orientalis* is also widespread in Asia [31,39,42] but less so than *C. tainanus* in Yunnan Province, China [38,43]. On the other hand, *C. obsoletus* is proven to be a BTV vector and was the most dominant species in Europe [44,45,46]. However, *C. obsoletus* contained a number of cryptic species that are difficult to discriminate morphologically [45,47,48]. Five species of the Obsoletus group, i.e., C. *obsoletus*, *Culicoides scoticus* Downes & Kettle, *Culicoides chiopterus* Meigen, *Culicoides dewulfi* Goetghebuer, and *Culicoides montanus* Shakirjanova, are found in Europe [49], while another species (*Culicoides albifascia* Tokunaga) has been described in Asia [50]. One or more *Culicoides* species belonging to the Obsoletus group have been found in several areas of Yunnan Province [31,51] and named Obsoletus. Although not the dominant species in Yunnan Province, they sometimes appear in cooler environments such as Shangri-La and Tengchong [31,51]. More research is needed to clarify these species in China.

A species closely related to *Culicoides pastus* Kitaoka, which was defined in Japan [3,7], was recently found in Yunnan Province, China [31,43]. However, this species, i.e., *C*. sp. nr *pastus* was not identified well until it was found to ideally match the morphological keys of *C. fenggangensis* which was defined in 2017 [52]. The only morphological difference between *C. fenggangensis* and *C.* sp. nr *pastus* is that *C. fenggangensis* has interfacial hairs on the ommateum [52] while *C.* sp. nr *pastus* does not (Figure S4A). However, we could not see interfacial hairs on their eyes in the photo in Chang’s article [52]. Based on the above and considering that there may be some misreads for a few detailed indices of *Culicoides* taxonomy between Chinese and occidental systems, we identified the so-called *C.* sp. nr *pastus* [31,43] as *C. fenggangensis* in the present study. In addition, a variant of *C. fenggangensis* (Figure 2) and a potential new species closely related to *C. fenggangensis* in both morphology and the *cox1* gene was found in this study and named *C.* sp. nr *fenggangensis* (Figure 2). This study represents the first publication of the *cox1* sequences of *C. fenggangensis* and the *C.* sp. nr *fenggangensis* (Table 3).

The subgenus *Hoffmania* was considered to be mainly restricted to locations below 1100 m a.s.l. [30]. However, at least 5 species, representing at least 2.3% of the collected midges, were collected in the present study in Tengchong (altitudes between 1375 and 2004 m a.s.l.). The dominant species were unidentified *Hoffmania* species with pale thoraces (color between faint brown and yellow). These *Hoffmania* species with faint brown thoraxes might be common in India [53], and one of them was confirmed as *C. regalis* by both morphology and *cox1* sequence (Figure 2 and Figure 3, Table 3) [53]. In addition, several *C. spiculae* and *C.* sp. nr *spiculae* were found. They all have spines on the cibarial armature (Figure S4B), but the wing pattern of *C.* sp. nr *spiculae* was somewhat different from that of typical *C. spiculae* [7] that the poststigmatic pale spot in cell R3 extends to the vein M1 for *C.* sp. nr *spiculae* (Figure 2). As their *cox1* sequences were highly homologous, *C.* sp. nr *spiculae* might be a variant or a subspecies of *C. spiculae*.

Normally, the midges of the subgenus *Trithecoides* were mainly restricted to hot and rainy areas such as south Yunnan Province (e.g., Puer and Xishuangbanna prefectures) [31] and Southeast Asia (e.g., Laos, Vietnam, Thailand, Malaysia) [7]. The numbers of *Trithecoides* collected in Tengchong, a relative cool area, was therefore unexpected. Among the *Trithecoides*, *C. palpifer* and *C. parahumeralis,* as well as *C.* sp. nr *palpifer* and *C.* sp. nr *parahumeralis* were the dominant species. The only morphological characteristic to distinguish species between *C. palpifer* and *C. parahumeralis* is that *C. parahumeralis* has three brown dots on the anterior margin of the mesonotum, which is absent in *C. palpifer* (Figure 3) [7]. *Culicoides* sp. nr *palpifer* was obviously different from *C. palpifer*, while *C.* sp. nr *parahumeralis* might be a variant of *C. parahumeralis*. Concretely, *C.* sp. nr *palpifer* has a similar wing pattern to *C. palpifer,* but its apical edge of the wing does not have a visible pale strip (Figure 2), and the features of the subapical pale band on the hind femur (Figure 3) and wrinkled spermatheca (Figure S4C) were similar as that of *Culicoides rugulithecus* Wirth & Hubert [7]. Typical *C. parahumeralis* has three discrete dark brown spots on the anterior margin of the mesonotum [7], while these spots became larger and contacted in *C.* sp. nr *parahumeralis* (Figure 3). In addition, *C.* sp. nr *rugulithecus* has similar morphology to *C. rugulithecus*, but its wings and abdomen were darker (Figure 2 and Figure 3).

Since *Culicoides marginus* Chu [54,55] was rare in China, not much is known about its potential as an arbovirus vector. In this study, we found *C.* sp. nr *marginus* and published its *cox1* and *28S* sequences for the first time (Table 3 and Table S3). The wing of *C. marginus* has a distinctive pale fork on the medial fork constructed by two lathy pale dots, which is inapparent in *C.* sp. nr *marginus* (Figure 2).

For the subgenus *Culicoides*, *C. newsteadi* (Asia), which is found widespread in East Asia, differs from *C. newsteadi* as found in Europe [56] and is identified as *C. punctatus* Latreille in some reports from Asia [41,43,57]. Meanwhile, another species, *C.* sp. (*Culicoides*), which was close to *C. nielamensis* Liu & Deng in morphology [58] and was discussed by us before [56], was rare in this investigation.

*Culicoides shortti* was first described in India in 1932 [3] and was common in Thailand [16,59]. In this study, a similar species was found and named *C.* sp. nr *shortti,* which has darker wings (Figure 2) than *C. shortti* [31,59]. This species was also recently found in Thailand [16].

Both *C. arakawae* and *C. arakawae* var were collected. The wing pattern of *C. arakawae* var is like that of *C. guttifer* [60], but in *C. guttifer*, the pale round dot under the radial cells never touches the radial veins. Although a total of 601 *C. arakawae* (including variants) were collected, there is no solid evidence that *C. arakawae* feeds on bovine, goats, or sheep, as only one specimen was blood-fed; this result is consistent with a previous study [56].

Based on our experience, bovine farms usually attract more *Culicoides* than goat farms in the same area, and that *Culicoides* abundance decreases as altitude increases. However, although many tiny insects were collected from the bovine farm (farm A), *Culicoides* accounted for approximately 5% of total insects, which resulted in the lowest collection of *Culicoides* among the three monitoring sites. Although the sheep farm (≥2000 m a.s.l.) had the highest altitude in this study, it yielded the biggest harvest of *Culicoides,* and the purity of *Culicoides* was ≥95% for the collections. This result suggested that the more developed the place, the fewer *Culicoides* appeared. In line with that, it is almost impossible to collect *Culicoides* around Kunming, the biggest city of Yunnan Province. Declines in terrestrial insects in anthropic zones have received increasing attention, often attributed to the destruction of natural insect habitats in some way [61,62,63]. Long-term light pollution at night and pesticide use may be important factors contributing to reduced midge populations in downtown areas.

Midges appeared between spring and mid-autumn and reached peak abundance between July and October. In addition, the three dominant potential BTV vectors (*C. tainanus*, Obsoletus, and *C. orientalis*) always maintained high rates of blood-fed + parous, which were hinted at a high risk of viral transmission. Cattle can act as circulating hosts for BTV [64], and these potential vectors shared between the cattle and sheep farms highlights the potential transmission of *Culicoides*-borne viruses such as BTV between these two hosts.

The present analysis suggested that a daily low temperature of 10 °C and an average rain score for the previous 10 days of 0.5 points are thresholds for adult midge activity, and low temperatures above 15 °C and scores above 1 point were ideal conditions for adult emergence. Accordingly, 16.5–17 °C was reported as the threshold temperature for the eclosion of *Culicoides brevitarsis* Kieffer pupae [65,66].

## 5. Conclusions

Tengchong County is in western Yunnan Province, China, and is located in the Sino-Burmese border area. It is warm and rainy in summer and cool and dry in winter. The diversity of *Culicoides* (at least 26 species) was relatively rich here, and the *C.* sp. nr *pastus* found in Yunnan Province before was reidentified as *C. fenggangensis*. The most dominant species on farm A (bovine) were Obsoletus (44.1%), *C. homotomus* (23.3%), and *C. arakawae* (12.9%); those on farm B (goats) were *C. tainanus* (68.0%), *C. orientalis* (12.6%), and *C. newsteadi* (Asia) (6.3%); and those on farm C (sheep) were *C. tainanus* (73.6%), *C. fenggangensis* (7.3%), and *C.* sp. nr *palpifer* (6.3%). A high number of potential cryptic species, collected at relative limited number of sites, indicated that an intensive taxonomic revision of the *Culicoides* species found in the area is required to support the present findings. The period between July and October would see a high incidence of epidemics of *Culicoides*-borne viruses in Tengchong. The relatively high *Culicoides* abundance and species richness indicate that this area is indeed at risk of the incursion of *Culicoides*-transmitted diseases. The movement of subclinical infected animals may initiate disease outbreaks in susceptible animals.

## Figures and Tables

**Table 1:** Light trap collection sites for *Culicoides* midges in Tengchong County of Yunnan Province, China, between May 2024 and April 2025.

Farm ID	Town	Major Animals	No. of Pens	Collection Date	Exact Coordinates		
					Latitude (°N)	Longitude (°E)	Elevation (m a.s.l.)
A	Teng-yue	30 bovine ^a^	1	2024–2025	25.062	98.542	1624
B	Bei-hai	30 goats	1	2024–2025	25.139	98.571	1794
C	Ming-guang	50 sheep	1	2024–2025	25.717	98.545	2004
D	Zhong-he	360 bovine ^a^	4	10 May 2024	25.032	98.371	1397
E	Zhong-he	80 goats	2	10 May 2024	25.021	98.394	1375

^a^ Cattle and Asian buffaloes; the numbers of cattle and buffaloes were roughly equal.

**Table 2:** *Culicoides* species collected by UV-traps at the three monitoring sites in Tengchong County of Yunnan Province, China, between May 2024 and April 2025.

Subgenus and Species	Total Number, Percentage, and Mean ± SD for Each Species			Frequency of Occurrences ^a^
	Farm A/Bovine (24 Collections)	Farm B/Goats (24 Collections)	Farm C/Sheep (20 Collections)	
*Avaritia*:	2125 (59.2%)	12,400 (86.0%)	63,155 (84.7%)	
*C. actoni*	0 (0.0%)	0 (0.0%)	0 (0.0%)	E (1)
*C. fenggangensis*	136 (3.8%) 5.7 ± 11.4	383 (2.7%) 16.0 ± 33.2	5475 (7.3%) 273.8 ± 379.8	all (38)
*C.* sp. nr *fenggangensis*	0 (0.0%)	3 (<0.1%) 0.1 ± 0.6	36 (<0.1%) 1.8 ± 5.2	ABC (5)
*C. hui*	0 (0.0%)	0 (0.0%)	1 (<0.1%) 0.1 ± 0.2	C (1)
*C. jacobsoni*	28 (0.8%) 1.2 ± 4.8	6 (<0.1%) 0.3 ± 1.2	0 (0.0%)	AB (3)
Obsoletus	1583 (44.1%) 66.0 ± 142.8	380 (2.6%) 15.8 ± 37.8	1112 (1.5%) 55.6 ± 129.6	ABCD (39)
*C. orientalis*	115 (3.2%) 4.8 ± 17.2	1821 (12.6%) 75.9 ± 205.6	1649 (2.2%) 82.5 ± 216.4	ABC (18)
*C. tainanus*	263 (7.3%) 11.0 ± 23.5	9807 (68.0%) 408.6 ± 887.9	54,882 (73.6%) 2744.1 ± 3865.6	all (45)
*Beltranmyia*:	0 (0.0%)	0 (0.0%)	0 (0.0%)	
*C. circumscriptus*	0 (0.0%)	0 (0.0%)	0 (0.0%)	E (1)
*Culicoides*:	48 (1.3%)	916 (6.4%)	571 (0.8%)	
*C. newsteadi* (Asia)	44 (1.2%) 1.8 ± 4.1	914 (6.3%) 38.1 ± 126.5	177 (0.2%) 8.9 ± 14.2	ABCD (33)
*C.* sp. (*Culicoides*)	4 (0.1%) 0.2 ± 0.6	2 (<0.1%) 0.1 ± 0.4	394 (0.5%) 19.7 ± 23.3	ABC (14)
*Hoffmania*:	58 (1.6%)	309 (2.1%)	1742 (2.3%)	
*C. parabubalus*	0 (0.0%)	1 (<0.1%) 0.0 ± 0.2	0 (0.0%)	B (1)
*C. regalis*	0 (0.0%)	0 (0.0%)	657 (0.9%) 32.9 ± 48.1	C (9)
*C. spiculae*	0 (0.0%)	3 (<0.1%) 0.1 ± 0.4	1 (<0.1%) 0.1 ± 0.2	BC (3)
*C.* sp. nr *spiculae*	0 (0.0%)	3 (<0.1%) 0.1 ± 0.4	44 (0.1%) 2.2 ± 5.5	BCD (6)
*C. sumatrae*	58 (1.6%) 2.4 ± 6.7	302 (2.1%) 12.6 ± 34.2	147 (0.2%) 7.4 ± 14.1	ABCE (19)
*Hoffmania* spp. (pale)	0 (0.0%)	0 (0.0%)	893 (1.2%) 44.7 ± 76.9	C (8)
*Meijerehelea*:	463 (12.9%)	20 (0.1%)	20 (<0.1%)	
*C. arakawae*	463 (12.9%) 19.3 ± 43.0	20 (0.1%) 0.8 ± 2.0	20 (<0.1%) 1.0 ± 4.4	ABCE (21)
*Monoculicoides*:	837 (23.3%)	0 (0.0%)	0 (0.0%)	
*C. homotomus*	837 (23.3%) 34.9 ± 52.2	0 (0.0%)	0 (0.0%)	A (13)
*Oecacta*:	2 (0.1%)	3 (<0.1%)	230 (0.3%)	
*C.* sp. nr *marginus*	2 (0.1%) 0.1 ± 0.4	3 (<0.1%) 0.1 ± 0.3	230 (0.3%) 11.5 ± 40.2	ABC (6)
*Remmia*:	1 (<0.1%)	0 (0.0%)	0 (0.0%)	
*C. oxystoma*	1 (<0.1%) 0.0 ± 0.2	0 (0.0%)	0 (0.0%)	A (1)
Shortti group:	0 (0.0%)	0 (0.0%)	5 (<0.1%)	
*C.* sp. nr *shortti*	0 (0.0%)	0 (0.0%)	5 (<0.1%) 0.3 ± 1.1	C (1)
*Trithecoides*:	56 (1.6%)	772 (5.4%)	8737(11.7%)	
*C. paksongi*	2 (0.1%) 0.1 ± 0.4	0 (0.0%)	126 (0.2%) 6.3 ± 11.2	AC (9)
*C. palpifer*	9 (0.3%) 0.4 ± 0.7	143 (1.0%) 6.0 ± 14.2	2148 (2.9%) 107.4 ± 200.2	ABCD (25)
*C.* sp. nr *palpifer*	0 (0.0%)	0 (0.0%)	4664 (6.3%) 233.2 ± 424.0	C (8)
*C. parahumeralis*	26 (0.7%) 1.1 ± 2.5	205 (1.4%) 8.5 ± 18.3	1310 (1.8%) 65.5 ± 142.8	all (24)
*C.* sp. nr *parahumeralis*	19 (0.5%) 0.8 ± 2.1	415 (2.9%) 17.3 ± 38.2	282 (0.4%) 14.1 ± 26.4	ABCD (25)
*C.* sp. nr *rugulithecus*	0 (0.0%)	2 (<0.1%) 0.1 ± 0.4	103 (0.1%) 5.2 ± 12.7	BCD (6)
*Trithecoides* spp.	0 (0.0%)	7 (<0.1%) 0.3 ± 0.9	104 (0.1%) 5.2 ± 10.1	BC (9)
Others ^b^	0 (0.0%)	3 (<0.1%) 0.1 ± 0.4	84 (0.1%) 4.2 ± 13.5	BCD (8)
Total	3590 (100.0%)	14,423 (100.0%)	74,544 (100.0%)	

^a^ The farms (farms A–E) where the corresponding category of *Culicoides* was found, and the numbers of collections (including two collections from farms D and E, respectively) containing this category. ^b^ Unclassified *Culicoides* specimens.

**Table 3:** The *cox1* sequences of *Culicoides* specimens collected by UV-traps in Tengchong County of Yunnan Province, China, between May 2024 and April 2025.

*Cox1* Sequence of *Culicoides* Specimen				Best Matched Record on NCBI ^a^
Specimen	Sample ID	BIN No.	Access No.	
*C. actoni*	YNS2-A6	AAJ7360	PV636443	*C. actoni*, KT352829.1 (99.9%)
*C. circumscriptus*	YNS2-A4	ACG0258	PV636441	*C. circumscriptus*, OM060694.1 (99.8%)
*C. circumscriptus*	YNS2-A5	ACG0258	PV636442	*C. circumscriptus*, OM060694.1 (97.7%)
*C. fenggangensis* ^b^	YNS2-A12	AEB1084	PV636446	*C.* sp., MF709270.1 (86.9%)
*C. fenggangensis* ^b^	YNS2-B10	AEB1084	PV690512	*C.* sp., KR672173.1 (86.8%)
*C.* sp. nr *fenggangensis* ^b^	YNS2-B8	NA	PV636449	*C. brevipalpis*, KT352210.1 (86.3%)
*C.* sp. nr *marginus* ^b^	YNS2-A7	AGH4070	PV636444	*C.* sp., JN302158.1 (84.5%)
*C.* sp. nr *marginus* ^b^	YNS2-A9	AGH4070	PV636445	*C.* sp., JN302158.1 (84.5%)
*C.* sp. nr *palpifer*	YNS2-B11	AFK8101	PV690513	*C.* sp., KM992425.1 (82.8%)
*C.* sp. nr *palpifer*	YNS2-B12	AFK8101	PV690514	*C.* sp., KM992425.1 (83.8%)
*C. regalis*	YNS2-B6	AFE7578	PV636448	*C. regalis*, OP405012.1 (94.6%)
*C. regalis*	YNS2-C6	AFE7578	PV690515	*C. regalis*, OP405012.1 (94.6%)
*C.* sp. nr *spiculae* ^b^	YNS2-B1	AFF5003	PV636447	*C. spiculae*, ON002362.1 (86.6%)

^a^ The species name, access number, and percent identity of base pairs for the best-matched record on NCBI. ^b^ *Culicoides* species whose *cox1* sequences were reported for the first time.

## Data Availability

The following information was supplied regarding the availability of DNA sequences: the new sequences are deposited in GenBank of NCBI under the accession numbers PV636441-PV636449, PV690512-PV690515, and PV643096-PV643099.

## Supplementary Materials

The following supporting information can be downloaded at https://www.mdpi.com/article/10.3390/insects16080780/s1, Table S1: Collections of *Culicoides* trapped by UV-traps at the monitoring points in Tengchong County of Yunnan Province, China, between May 2024 and April 2025; Table S2: Amounts of *Culicoides* species collected by UV-traps at farms D and E in Tengchong County of Yunnan Province, China, between May 2024 and April 2025; Table S3: The *28S* sequences of *Culicoides* specimens collected by UV-traps in Tengchong County of Yunnan Province, China, between May 2024 and April 2025; Figure S1. Examples of the statuses of *Culicoides*. The status of male (A), blood-fed (B), parous (C), and nulliparous (D) were shown using *C. tainanus* collected in this study. (E) A mounted abdomen of gravid *Culicoides morisitai* Tokunaga collected from Lufeng County previously, it was full of eggs. Critical parts were marked by arrows: (a) clustered long hairs; (b) male genitalia; (c) blood meal in abdomens; (d) pigmentation on the inner side of abdomen; (e) abdomens without pigment. Scale bar = 0.5 mm; Figure S2: The status composition of *Culicoides* Obsoletus collected by UV-traps in Tengchong County of Yunnan Province, China, between May 2024 and April 2025. The numbers of the four midge categories (males, parous/gravid females, blood-fed females, and nulliparous females) of Obsoletus in every collection from the three monitoring sites (A, B, and C) were shown; Figure S3: The status composition of *C. orientalis* collected by UV-traps in Tengchong County of Yunnan Province, China, between May 2024 and April 2025. The numbers of the four midge categories (males, parous/gravid females, blood-fed females, and nulliparous females) of *C. orientalis* in every collection from the three monitoring sites were shown; Figure S4: Detailed photos under a 40x objective lens. Samples were collected by UV-traps in Tengchong County of Yunnan Province, China, between May 2024 and April 2025. (A) The compound eye of *C.* sp. nr *pastus* (=*C. fenggangensis*, sample ID: YNS2-A12) and four blue arrows (a–d) pointing out the interfacial spaces: space a seems to have hairs, but spaces b, c, and d do not have hair. (B) The cibarial armature of *C.* sp. nr *spiculae* (sample ID: YNS2-B1) and the spines are pointed to by a red arrow. (C) The spermatheca of *C.* sp. nr *palpifer* (sample ID: YNS2-B11); Supplementary S1: The effects of high, mid, and low temperatures and average rainfall of 2, 5, 10 days before collecting on newborn *Culicoides* collected by UV-traps in Tengchong County of Yunnan Province, China, between May 2024 and April 2025.
