# Supplementary material for: Diversity and Seasonal Abundance of Culicoides (Diptera: Ceratopogonidae) in Tengchong County of Yunnan, China
Source: Insects. 2025 Jul 30;16(8):780. doi: 10.3390/insects16080780 (PMC12386476; doi:10.3390/insects16080780)
Supplement: Supplementary file 1 [file insects-16-00780-s001.zip › Table S1=ABC collections R2.pdf]

Table S1. Collections of *Culicoides* trapped by UV-traps at the monitoring points in Tengchong County of Yunnan Province, China, between May 2024 and April 2025.

Batch	Month/season	Farm A (bovine)			Farm B (goats)			Farm C (sheep)		
		Collection Date	Amount	Mean value (mean ± SD)	Collection Date	Amount	Mean value (mean ± SD)	Collection Date	Amount	Mean value (mean ± SD)
1	May 2024	11 May 2024	121	149.5 ± 28.5	11 May 2024	27	67.0 ± 40.0	NA	NA	NA
2		28 May 2024	178		28 May 2024	107		NA	NA	
3	Jun 2024	12 Jun 2024	116	124.5 ± 8.5	12 Jun 2024	438	254.0 ± 184.0	NA	NA	17,440.0 ± 0.0
4		26 Jun 2024	133		26 Jun 2024	70		04 Jul 2024	17,440	
5	Jul 2024	12 Jul 2024	222	577.0 ± 355.0	12 Jul 2024	3,910	4,460.0 ± 550.0	NA	NA	6,240.0 ± 0.0
6		26 Jul 2024	932		26 Jul 2024	5,010		01 Aug 2024	6,240	
7	Aug 2024	13 Aug 2024	260	197.0 ± 63.0	13 Aug 2024	976	887.0 ± 89.0	15 Aug 2024	4,950	8,782.5 ± 3,832.5
8		29 Aug 2024	134		29 Aug 2024	798		02 Sep 2024	12,615	
9	Sep 2024	12 Sep 2024	550	399.5 ± 150.5	12 Sep 2024	304	724.0 ± 420.0	14 Sep 2024	8,057	8,493.5 ± 436.5
10		01 Oct 2024	249		30 Sep 2024	1,144		30 Sep 2024	8,930	
11	Oct 2024	18 Oct 2024	30	237.5 ± 207.5	18 Oct 2024	199	387.5 ± 188.5	18 Oct 2024	314	2,112.0 ± 1,798.0
12		31 Oct 2024	445		31 Oct 2024	576		31 Oct 2024	3,910	
13	Nov 2024	18 Nov 2024	2	1.0 ± 1.0	15 Nov 2024	11	7.5 ± 3.5	15 Nov 2024	128	64.0 ± 64.0
14		02 Dec 2024	0		02 Dec 2024	4		02 Dec 2024	0	
15	Dec 2024	16 Dec 2024	0	4.5 ± 4.5	16 Dec 2024	1	0.5 ± 0.5	16 Dec 2024	0	0.0 ± 0.0
16		31 Dec 2024	9		31 Dec 2024	0		01 Jan 2025	0	
17	Jan 2025	16 Jan 2025	0	0.0 ± 0.0	16 Jan 2025	0	0.0 ± 0.0	16 Jan 2025	0	0.0 ± 0.0
18		27 Jan 2025	0		27 Jan 2025	0		27 Jan 2025	0	
19	Feb 2025	17 Feb 2025	0	23.0 ± 23.0	17 Feb 2025	0	0.0 ± 0.0	17 Feb 2025	63	266.5 ± 203.5
20		03 Mar 2025	46		03 Mar 2025	0		03 Mar 2025	470	

21	Mar 2025	17 Mar 2025	98	49.0 ± 49.0	17 Mar 2025	0	9.0 ± 9.0	17 Mar 2025	2,640	1,336.0 ± 1,304.0
22		02 Apr 2025	0		02 Apr 2025	18		02 Apr 2025	32	
23	Apr 2025	17 Apr 2025	0	32.5 ± 32.5	17 Apr 2025	66	415.0 ± 349.0	17 Apr 2025	6,430	4,377.5 ± 2,052.5
24		30 Apr 2025	65		30 Apr 2025	764		30 Apr 2025	2,325	
**Total**			**3,590**			**14,423**			**74,544**	
1 - 2, 21 - 24	Spring			77.0 ± 64.0			163.7 ± 270.7			2,856.8 ± 2,295.5
3 – 8	Summer			299.5 ± 287.6			1,867.0 ± 1,882.4			10,311.3 ± 5,036.0
9 - 14	Autumn			212.7 ± 220.6			373.0 ± 395.4			3,556.5 ± 3,745.5
15 - 20	Winter			9.2 ± 16.8			0.2 ± 0.4			88.8 ± 172.0
**4 - 12**	**Peak season**			**328.3 ± 261.3**			**1,443.0 ± 1,667.2**			**7,807.0 ± 4,989.8**
