# Supplementary material for: Diversity and Seasonal Abundance of Culicoides (Diptera: Ceratopogonidae) in Tengchong County of Yunnan, China
Source: Insects. 2025 Jul 30;16(8):780. doi: 10.3390/insects16080780 (PMC12386476; doi:10.3390/insects16080780)
Supplement: Supplementary file 1 [file insects-16-00780-s001.zip › Table S2=DE collections proofread.pdf]

Table S2. Amounts of *Culicoides* species collected by UV-traps at farms D and E in Tengchong County of Yunnan Province, China, between May 2024 and April 2025.

Subgenus	Species	Farm D (bovine)	Farm E (goats)
Avaritia	*C. actoni*	0	1
	C. fenggangensis	3	12
	Obsoletus	2	0
	C. tainanus	17	19
Beltranmyia	*C. circumscriptus*	0	2
Culicoides	*C. newsteadi* (Asia)	1	0
Hoffmania	*C.* sp nr *spiculae*	1	0
	C. sumatrae	0	2
Meijerehelea	*C. arakawae*	0	98
Trithecoides	*C. palpifer*	19	0
	C. parahumeralis	72	2
	C. sp. nr *parahumeralis*	19	0
	C. sp. nr *rugulithecus*	2	0
Uncertain	Others	1	0
**Total**		**137**	**136**
