# Supplementary material for: Diversity and Seasonal Abundance of Culicoides (Diptera: Ceratopogonidae) in Tengchong County of Yunnan, China
Source: Insects. 2025 Jul 30;16(8):780. doi: 10.3390/insects16080780 (PMC12386476; doi:10.3390/insects16080780)
Supplement: Supplementary file 1 [file insects-16-00780-s001.zip › Table S3=28S Seq proofread.pdf]

Table S3. The *28S* sequences of *Culicoides* specimens collected by UV-traps in Tengchong County of Yunnan Province, China, between May 2024 and April 2025.

28S sequence of *Culicoides* specimen			Best matched record on NCBI	
Specimen	Sample ID	Access No.	Close species [a]	Access No.
C. sp. nr *fenggangensis* [b]	YNS2-B8	PV643099	*C. gulbenkiani* (97.0%)	MF423028.1
C. sp. nr *marginus* [b]	YNS2-A7	PV643096	*C. ovalis* (97.1%)	MF423082.1
C. sp. nr *marginus* [b]	YNS2-A9	PV643097	*C. ovalis* (97.0%)	MF423082.1
C. regalis	YNS2-B6	PV643098	*C. sumatrae* (96.0%)	OM655252.1

a. The species name and percent identity of base pairs for the best-matched record on NCBI.

b. *Culicoides* species whose *28S* sequences were reported for the first time.
