# Supplementary figures and images for: Diversity and Seasonal Abundance of Culicoides (Diptera: Ceratopogonidae) in Tengchong County of Yunnan, China
Source: Insects. 2025 Jul 30;16(8):780. doi: 10.3390/insects16080780 (PMC12386476; doi:10.3390/insects16080780)

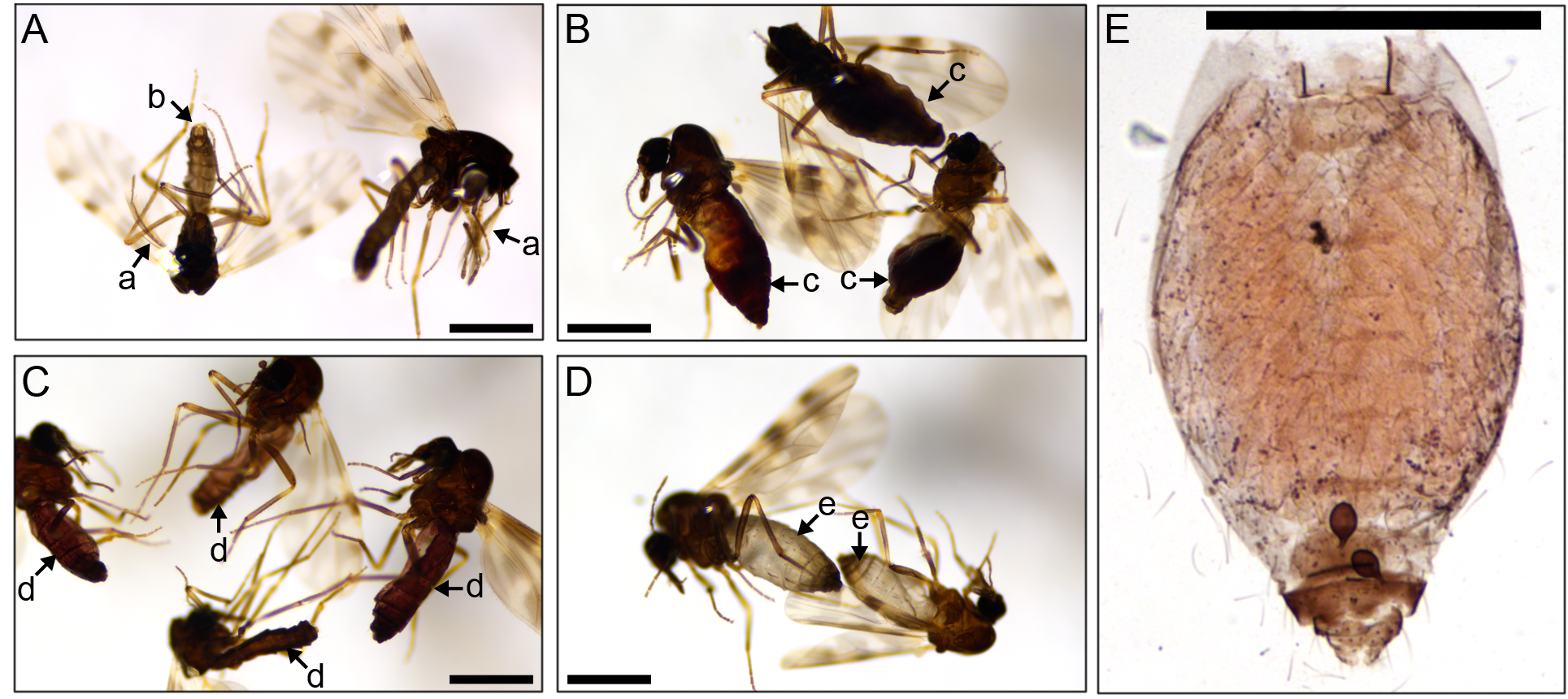

Supplement: Supplementary file 1 [file insects-16-00780-s001.zip › Figure S1=Status R1.png]

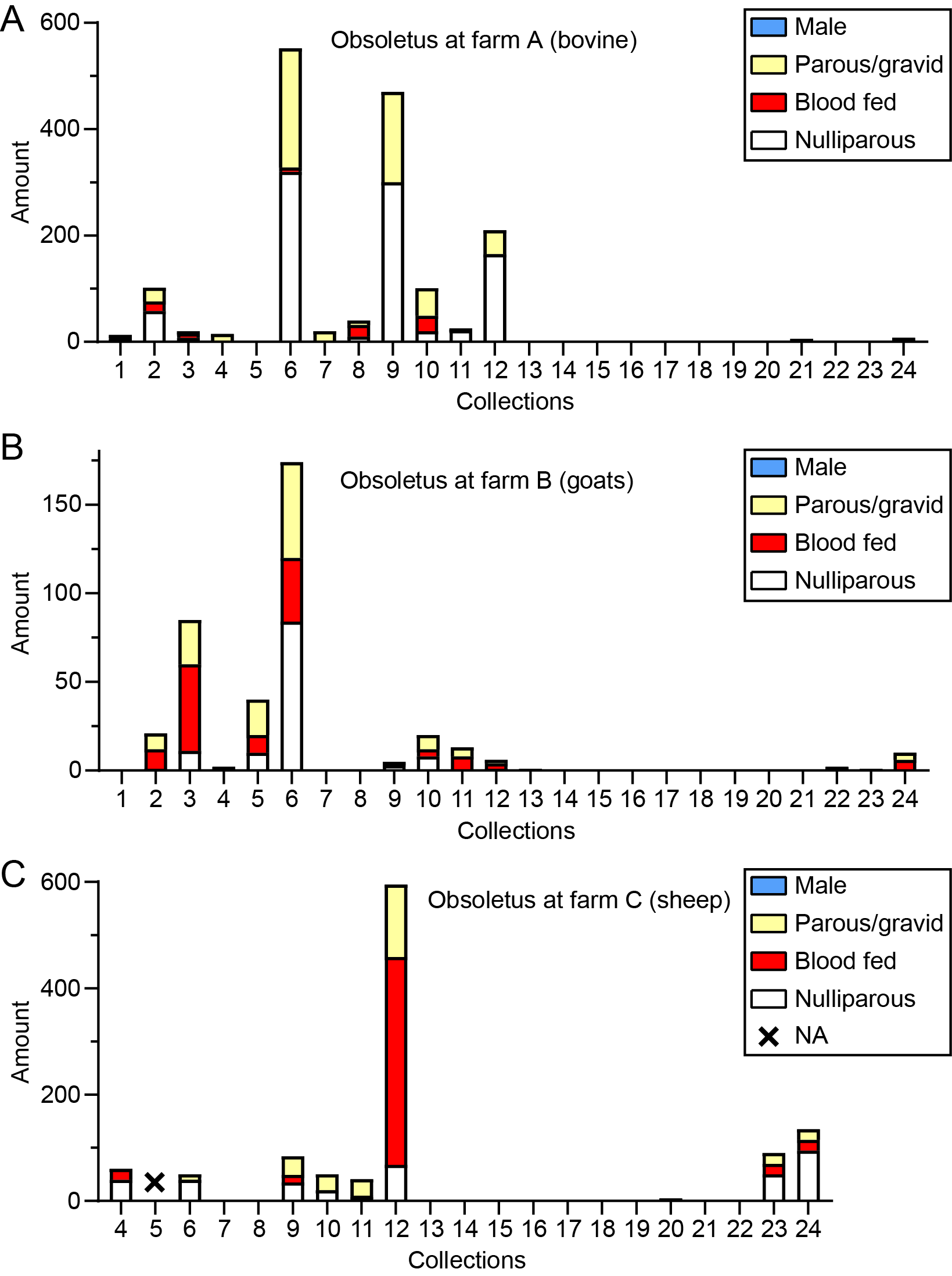

Supplement: Supplementary file 1 [file insects-16-00780-s001.zip › Figure S2=Obsoletus R1.png]

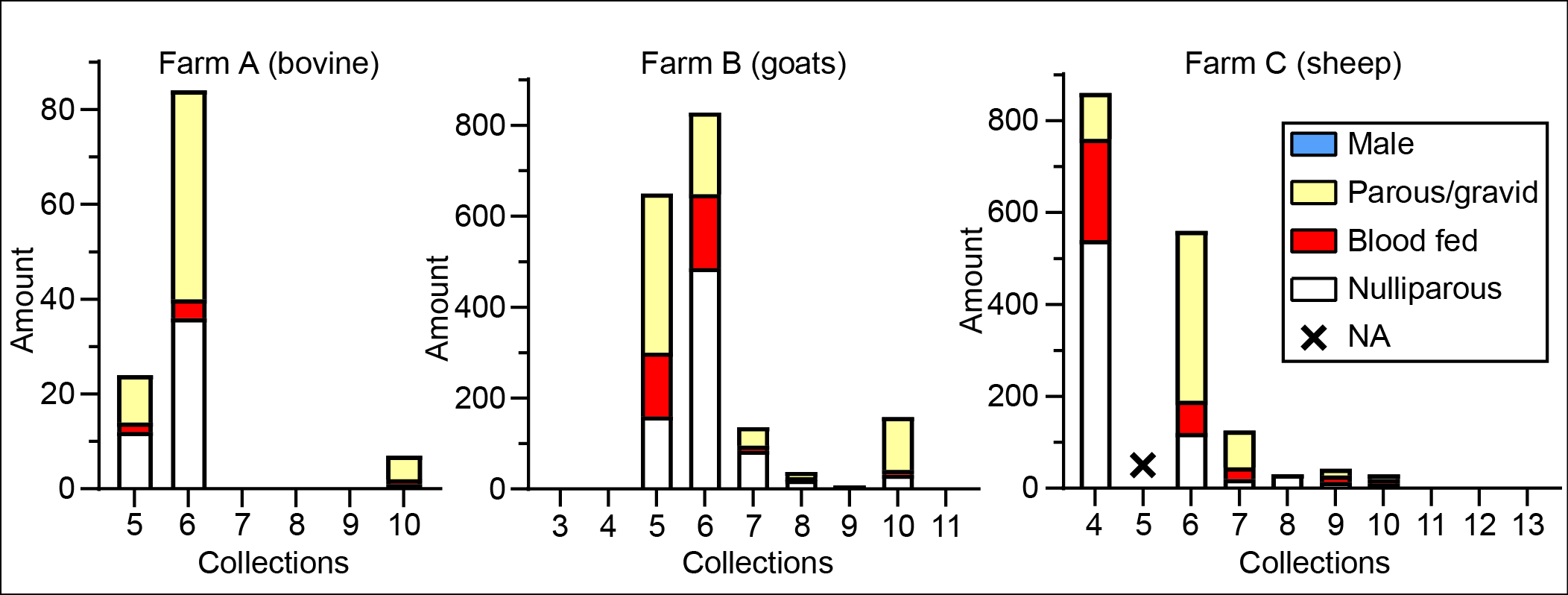

Supplement: Supplementary file 1 [file insects-16-00780-s001.zip › Figure S3=orientalis R1.png]

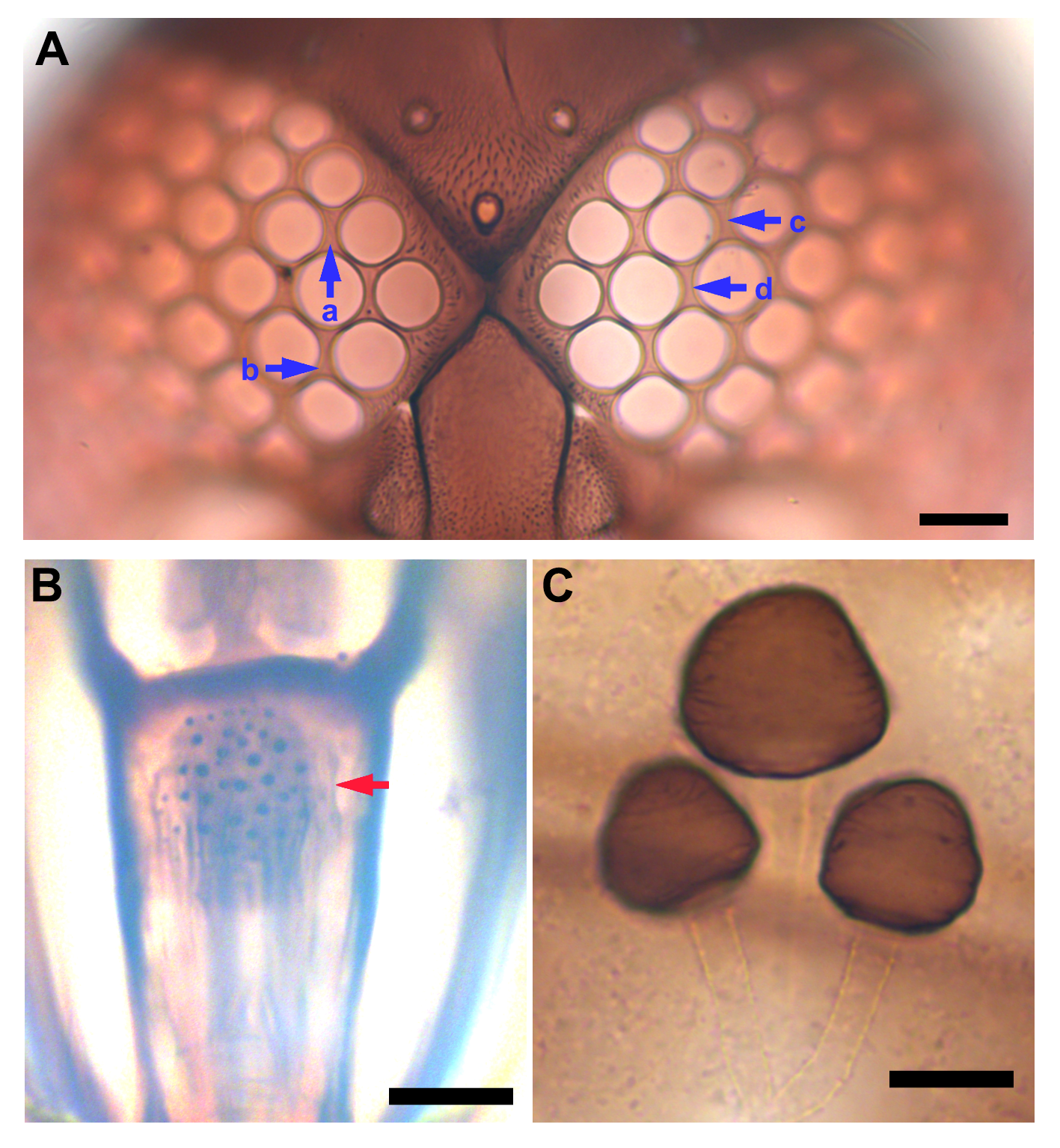

Supplement: Supplementary file 1 [file insects-16-00780-s001.zip › Figure S4=40x photos R1.tif]

# Bovine farms (farms A and D)

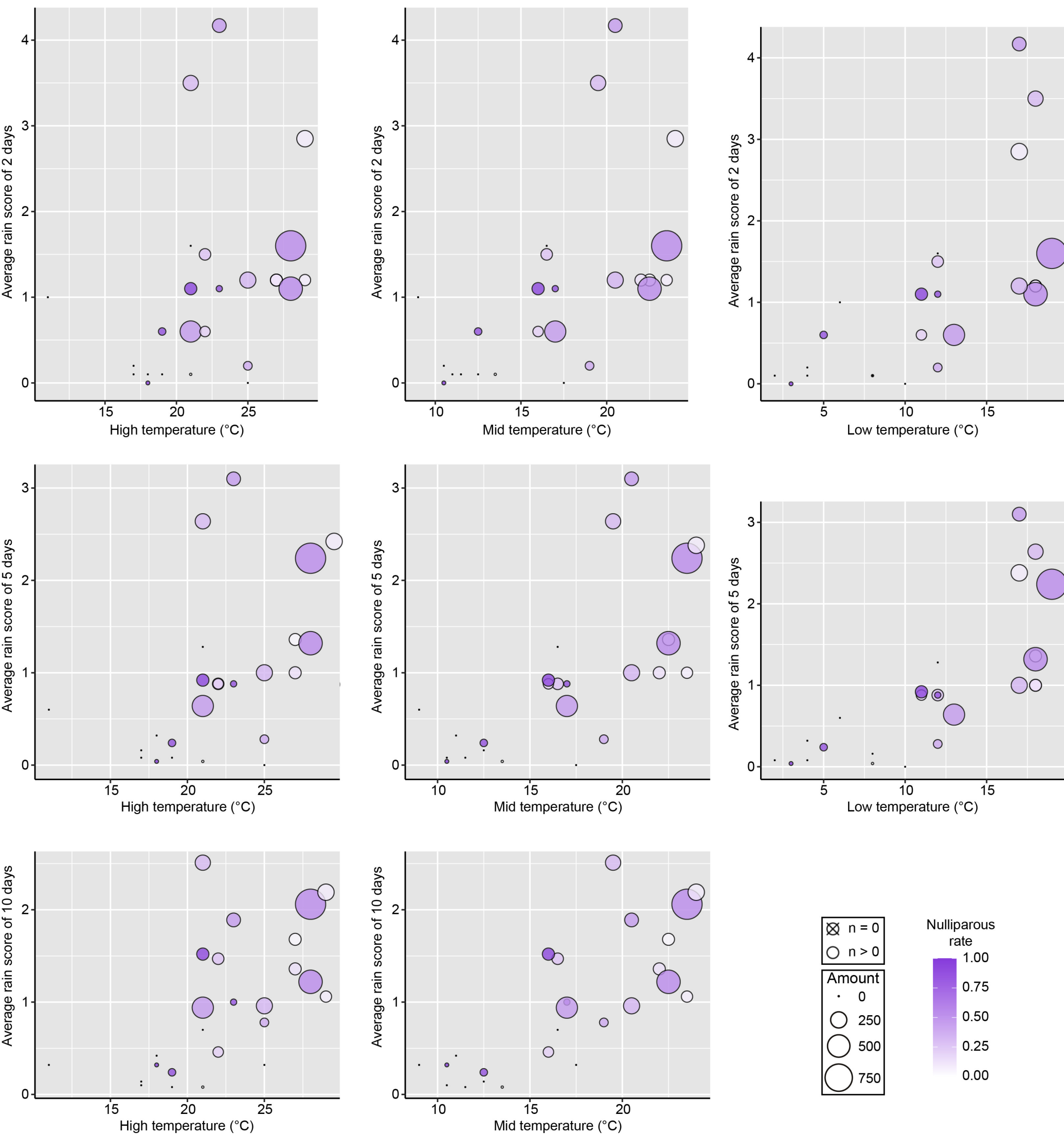

# Goat farms (farms B and E)

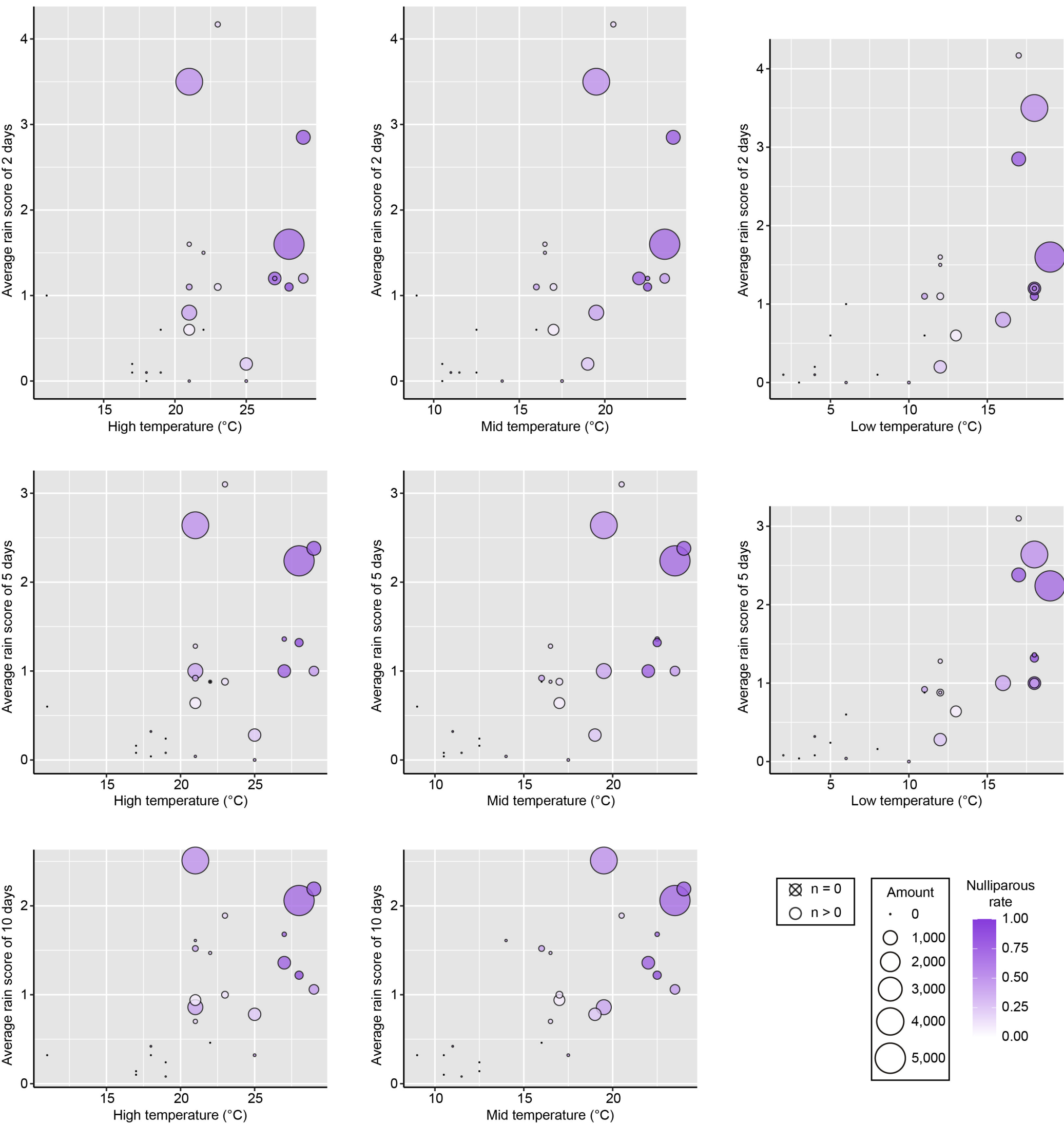

# Sheep farm (farm C)

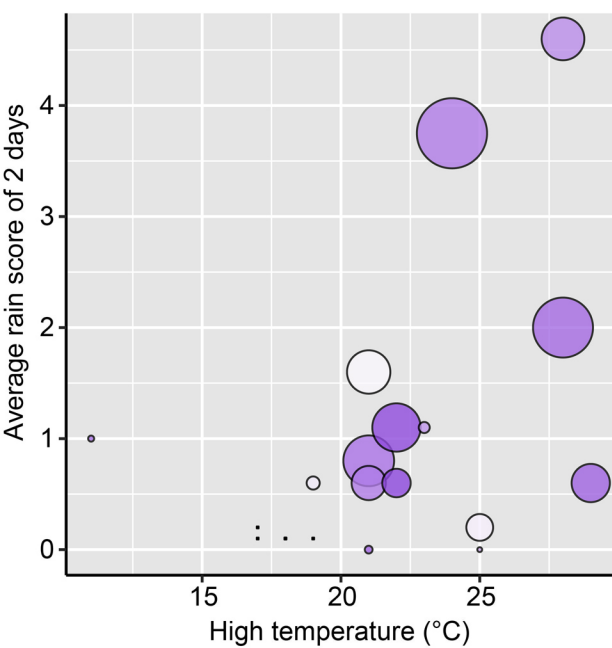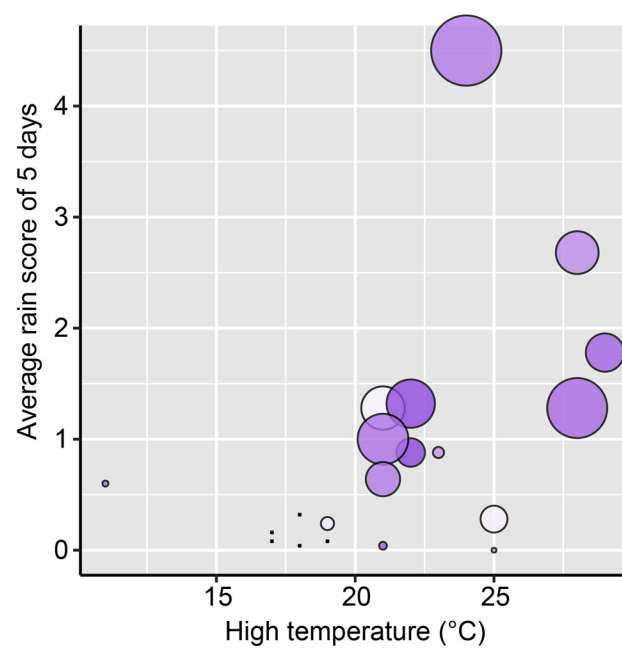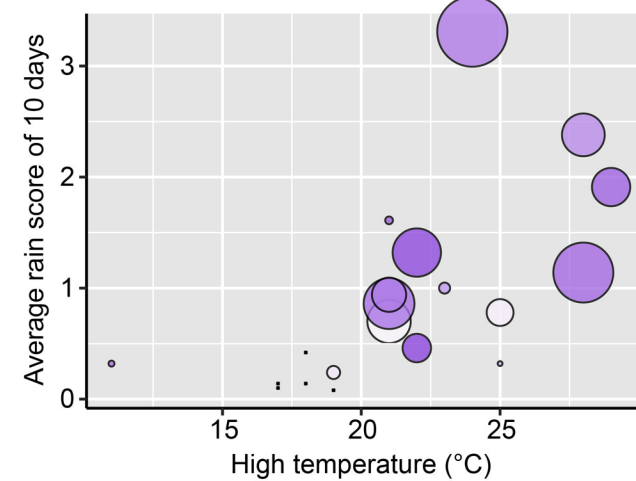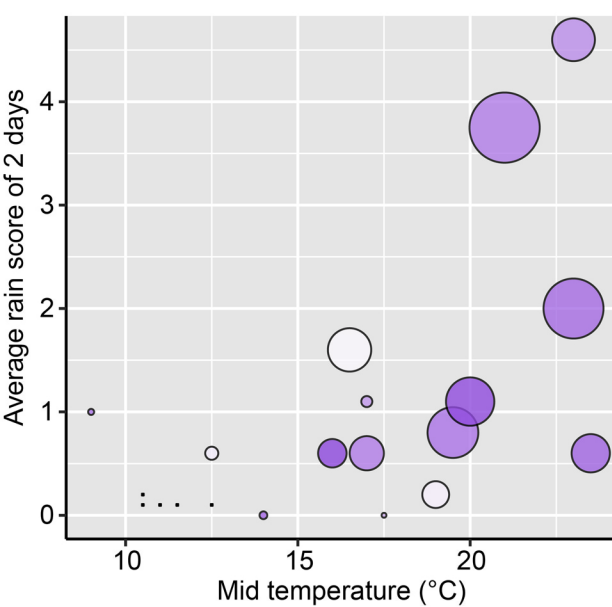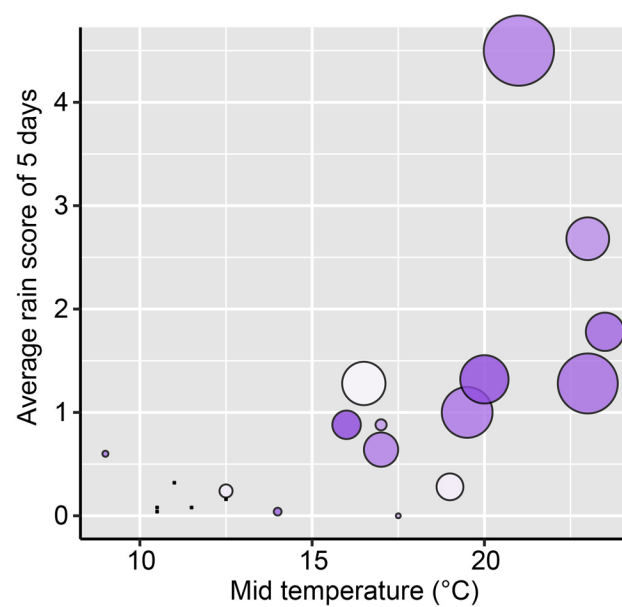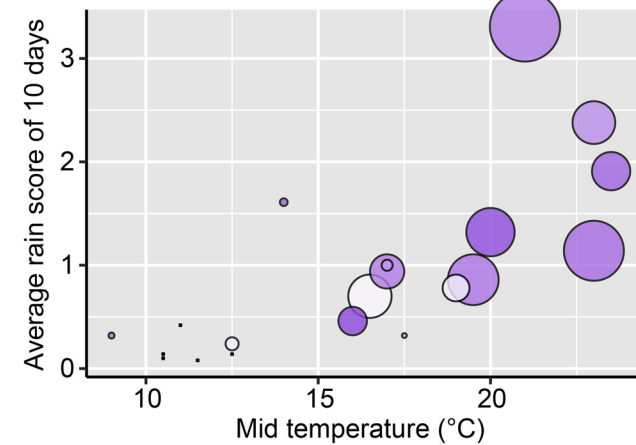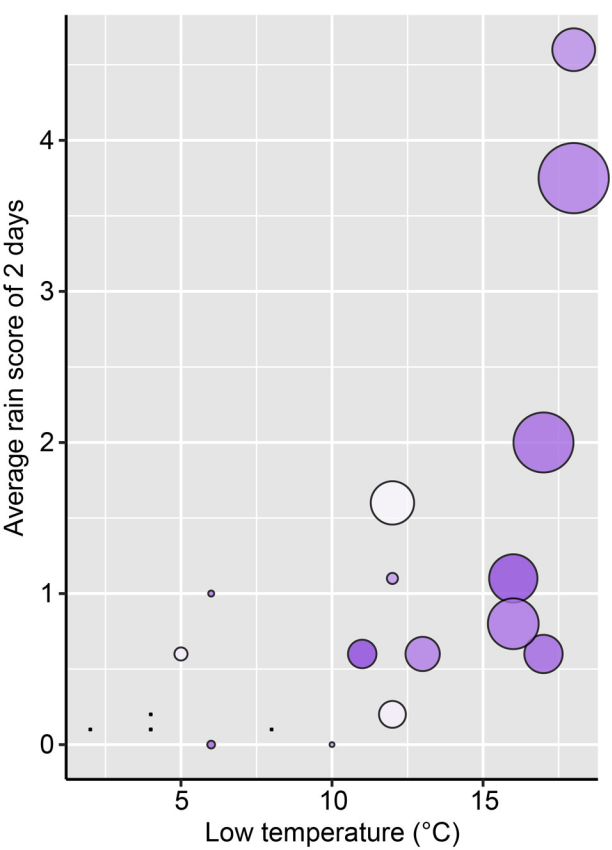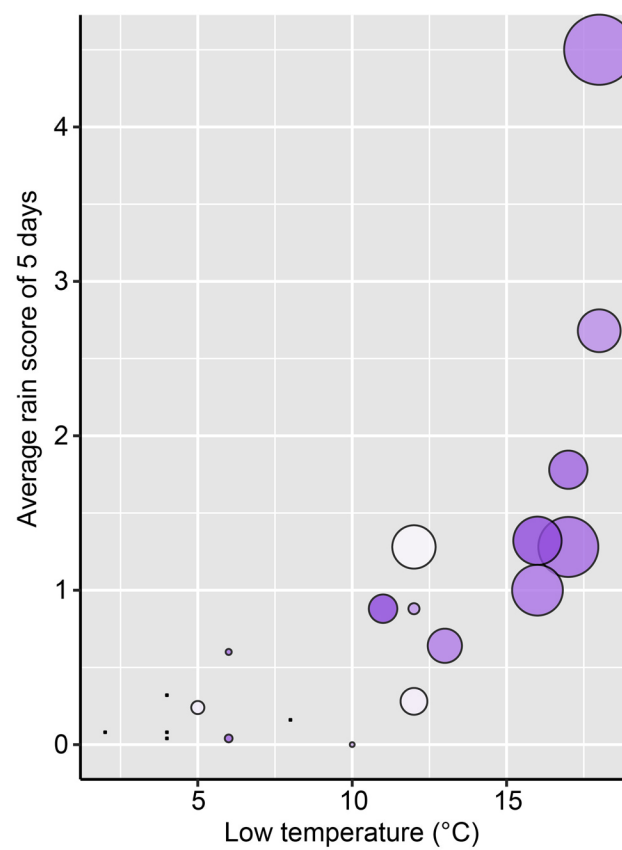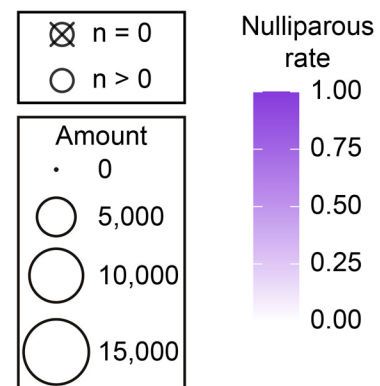

Supplement: Supplementary file 1 [file insects-16-00780-s001.zip › Supplementary S1.pdf]
